# Recovery from severe H7N9 disease is associated with diverse response mechanisms dominated by CD8^+^ T cells

**DOI:** 10.1038/ncomms7833

**Published:** 2015-05-13

**Authors:** Zhongfang Wang, Yanmin Wan, Chenli Qiu, Sergio Quiñones-Parra, Zhaoqin Zhu, Liyen Loh, Di Tian, Yanqin Ren, Yunwen Hu, Xiaoyan Zhang, Paul G. Thomas, Michael Inouye, Peter C. Doherty, Katherine Kedzierska, Jianqing Xu

**Affiliations:** 1 Shanghai Public Health Clinical Center and Institutes of Biomedical Sciences, Key Laboratory of Medical Molecular Virology of Ministry of Education/Health, Shanghai Medical College, Fudan University, Shanghai 201508, China; 2 Department of Microbiology and Immunology, University of Melbourne, at the Peter Doherty Institute for Infection and Immunity, Parkville 3010, Victoria, Australia; 3 Department of Immunology, St Jude Children’s Research Hospital, Memphis, Tennesse 38105, USA; 4 Medical Systems Biology, Department of Pathology, University of Melbourne, Parkville, 3010, Australia

## Abstract

The avian origin A/H7N9 influenza virus causes high admission rates (>99%) and mortality (>30%), with ultimately favourable outcomes ranging from rapid recovery to prolonged hospitalization. Using a multicolour assay for monitoring adaptive and innate immunity, here we dissect the kinetic emergence of different effector mechanisms across the spectrum of H7N9 disease and recovery. We find that a diversity of response mechanisms contribute to resolution and survival. Patients discharged within 2–3 weeks have early prominent H7N9-specific CD8^+^ T-cell responses, while individuals with prolonged hospital stays have late recruitment of CD8^+^/CD4^+^ T cells and antibodies simultaneously (recovery by week 4), augmented even later by prominent NK cell responses (recovery >30 days). In contrast, those who succumbed have minimal influenza-specific immunity and little evidence of T-cell activation. Our study illustrates the importance of robust CD8^+^ T-cell memory for protection against severe influenza disease caused by newly emerging influenza A viruses.

## Abstract (web summary)

H7N9 avian influenza viruses can cause severe human disease. Here, the authors analyse blood samples from hospitalized H7N9 patients and show that a diversity of immune mechanisms seem to influence disease length and outcome.

## Graphical abstract

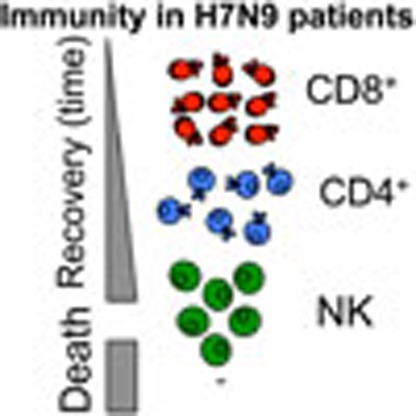

Since March 2013, the novel A/H7N9 avian-derived influenza A virus (AIAV) has caused 602 human cases, with a 38% mortality. Most (>99%) H7N9-infected individuals were hospitalized with severe pneumonia (97.3%) and acute respiratory distress syndrome (71.2%), leading to high rates of ICU admissions (75%) and mechanical ventilation (66%)^1^,^2^. Manifestations also included multiorgan failure and early hypercytokemia^3^,^4^,^5^, driven at least partially by the IFITM3 (ref. 4) host genetic factor. The majority had contact with poultry, although possible person-to-person spread is suggested by ferret experiments^6^ and evidence of transmission from close family contact^7^. The need to understand H7N9 pathogenesis and immunity is highlighted by (i) the prevalence of severe H7N9 cases (602 over two years versus 652 caused by the H5N1 AIAV over 10 years), (ii) the possibility that mutation could facilitate human-to-human spread^8^ and (iii) that further reassortment with H9N2 AIAVs could lead to the emergence of a more transmissible strain.

In the absence of neutralizing antibodies (NAbs) to newly-emerged IAVs, pre-existing CD8^+^ or CD4^+^ T-cell memory promotes recovery from experimentally or naturally mild H3N2 and H1N1-2009 IAV infections^9^,^10^,^11^,^12^. However, while extensively studied in mouse models, the kinetics and role of CD8^+^ and/or CD4^+^ T-cell responses in human influenza (especially when severe) is far less clear. Furthermore, such data are conspicuously absent for severe A/H7N9 or A/H5N1 infections in antibody-naive individuals. Understanding the immune mechanisms central to recovery from novel IAV exposure is an urgent need.

In 2013, we collected longditudinal peripheral blood mononuclear cell (PBMC) samples from 16 hospitalized H7N9 patients^4^,^13^,^14^ for retrospective analysis. Here we analyse these samples using a novel 13-colour method that quantifies H7N9-specific CD8^+^ cytotoxic T lymphocyte (CTL) and CD4^+^ T helper (T_H_) frequencies together with measures of innate NK cells, γδ T cells, mucosal-associated invariant T cells (MAITs) and monocytes. Correlation with antibody titres, virus titration, immune gene expression profiling and symptoms provides new insights into the nexus between host response and recovery, including evidence that a diversity of immune mechanisms influence disease length and outcome. Patients recovering within 3 weeks of clinic onset had rapid and robust CTL recall responses followed by antibody detection within a further 2–3 days (d). Those who were ill for a longer time (to week 4) had IAV-specific CTL responses later, with the T_H_ component emerging more strongly, along with NK cells in the >30d recovery group. Fatal outcomes were associated with a diminution in all responses. Different components of immunity thus seem to be sequentially recruited, depending on the duration of severe H7N9 disease, with early CTL emergence providing optimal protection.

## Results

### Patient demographics and study design

Immune effectors were quantified for 16 H7N9 patients (confirmed by using PCR^4^) hospitalized during the first wave of an outbreak. Twelve patients recovered between d14 and d35, while the remainder succumbed. Blood was obtained for one or more tests at 3–4d intervals and symptoms were closely monitored. Viral RNA was monitored from throat swabs, blood, stools and urine using real-time PCR. Most patients were admitted at ∼d7 after the disease onset, and viral RNA was detected for the majority on d8–d14, contrasting with earlier studies of human A/H3N2 and A/H1N1pdm09 infections which were negative by d7^11^,^15^,^16^. Patient demographics (age, human leukocyte antigen (HLA) typing, days of hospitalization/clinical onset, oseltamivir and comorbidities) are provided in Supplementary Table 1. There were no significant differences between the survival and fatal groups in age, gender, co-medical conditions, time from disease onset to oseltamivir treatment, time from disease onset to hospitalization or between a number of admission days after disease onset and the length of hospital stay (Supplementary Figs 1 and 2). However, it is worth noting that two out of four H7N9 fatal cases had a coexisting medical condition of chronic bronchitis, a mild chronic obstructive pulmonary (COPD). Those two patients (a131 and a118) had only mild symptoms of COPD, as they did not require corticosteroid treatment to attenuate their symptoms before H7N9 hospital admission. Indeed, both patients had only received one dose of Methylprednisolone (a131 on day 6 and a118 on day 8 after the onset of illness) for treatment during their hospitalization. Similarly, eight survivors received one dose of Methylprednisolone, while one survivor received two doses. Therefore, although we cannot exclude the possibility that their mild COPD might have contributed to their fatal outcome since influenza infection, it is unlikely that administration of Methylprednisolone significantly interfered with their immune responses in these two subjects but not in the other eight survival patients. Furthermore, two of the oldest patients (aged 79 and 88) died, although patients of similar ages (74, 74, 75 and 78) were also in the ‘survival’ group.

All patients with fatal clinical outcomes died of respiratory failure. Three patients, a22, a33 and a118, developed severe respiratory failure and received ECMO (extracorporeal membrane oxygenation) within 2–3 weeks after their admission to hospital, but unfortunately, although ECMO extended their lives, it was unable to regain their lung function. One patient (a131) developed respiratory failure early and received mechanical ventilator on day 3 after hospital admission, but died of aggravated respiratory failure likely due to hospital-acquired co-infection of bacteria during the prolonged hospitalization.

Immune analysis of PBMCs used a 13-colour, interferon (IFN)-γ-based flow cytometric assay that quantifies H7N9-specific CD8^+^ CTLs and CD4^+^ T_H_ cells, NK cells, γδ T cells and MAIT cells following incubation with the H7N9 virus and subsequent 18-h cell culture (Supplementary Fig. 3). The reproducibility of the assay was verified using replicates at multiplicity of infection (MOI) of 4 (Supplementary Fig. 3c).

### Elevated cellular responses in the recovery patient group

**Figure 1:**
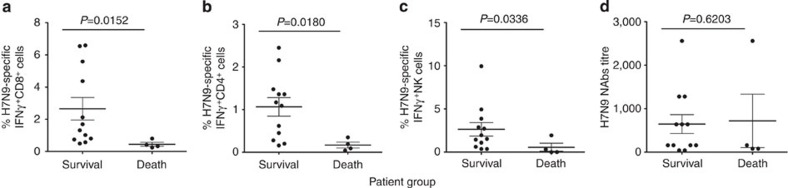
Elevated IFN-γ-producing CD8^+^, CD4^+^ and NK responses in the H7N9 recovery patient group. H7N9-specific (**a**) CD8^+^, (**b**) CD4^+^ and (**c**) NK cells but not (**d**) NAbs are significantly increased in patients who survived (*n*=12) when compared with individuals who died (*n*=4) from the severe influenza disease, as detected by IFN-γ ICS following 18-h stimulations with A/H7N9 virus. The comparison of the highest numbers of CD8^+^, CD4^+^, NK and NAbs during the disease course of each patient between survival and fatal groups was performed using Mann–Whitney test.

The main goal was to dissect immune recovery mechanisms in severe H7N9 disease. Overall comparison of immune effectors in patients who survived (*n*=12) and patients who died (*n*=4) showed that the recovery patient group had significantly higher H7N9-specific IFN-γ-producing CD8^+^, CD4^+^ and NK cell immune responses (Fig. 1; *P*<0.05). Importantly, this also means that fatal H7N9 disease outcomes were associated with reduced cellular responses in comparison with the survival patient group. As our hypothesis was that effector immune responses were inversely related to the severity and duration of severe H7N9 disease, we further analysed immunity for the recovery versus death groups in relation to the disease length and outcome. Our careful dissection of immune responses in the individual patients in the ‘recovery’ group showed that the longer the hospitalization with severe H7N9 disease was, the more diverse the immune responses became, involving first CD8^+^ CTLs, and then CD4^+^ T_H_ cells, NAbs, and in those that recovered late, the emergence of prominent, innate NK cell populations. To further visualize these interesting observations, the patients were divided into three recovery groups on the basis of illness duration (hospital stay length) and disease outcome: R1 (d14–d18), R2 (d21–27), R3 (d31–35) and RD (fatal outcome; Table 1).

### Early CTL responses are associated with rapid recovery

Expecting to see CTL responses within 10 days of onset^12^,^17^, we focused (where available) on samples from ∼d10 (d9–d12), ∼d21 (d19–d23) and ∼d30 (d29–d30). Apart from those concurrent analyses, we later stimulated PBMCs from earlier time points for selected patients with H7N9 virus (a107 and a49 from R1, a134 and a130 from R2, a11 and a12 from R3 and a22 and a33 from RD).

**Figure 2:**
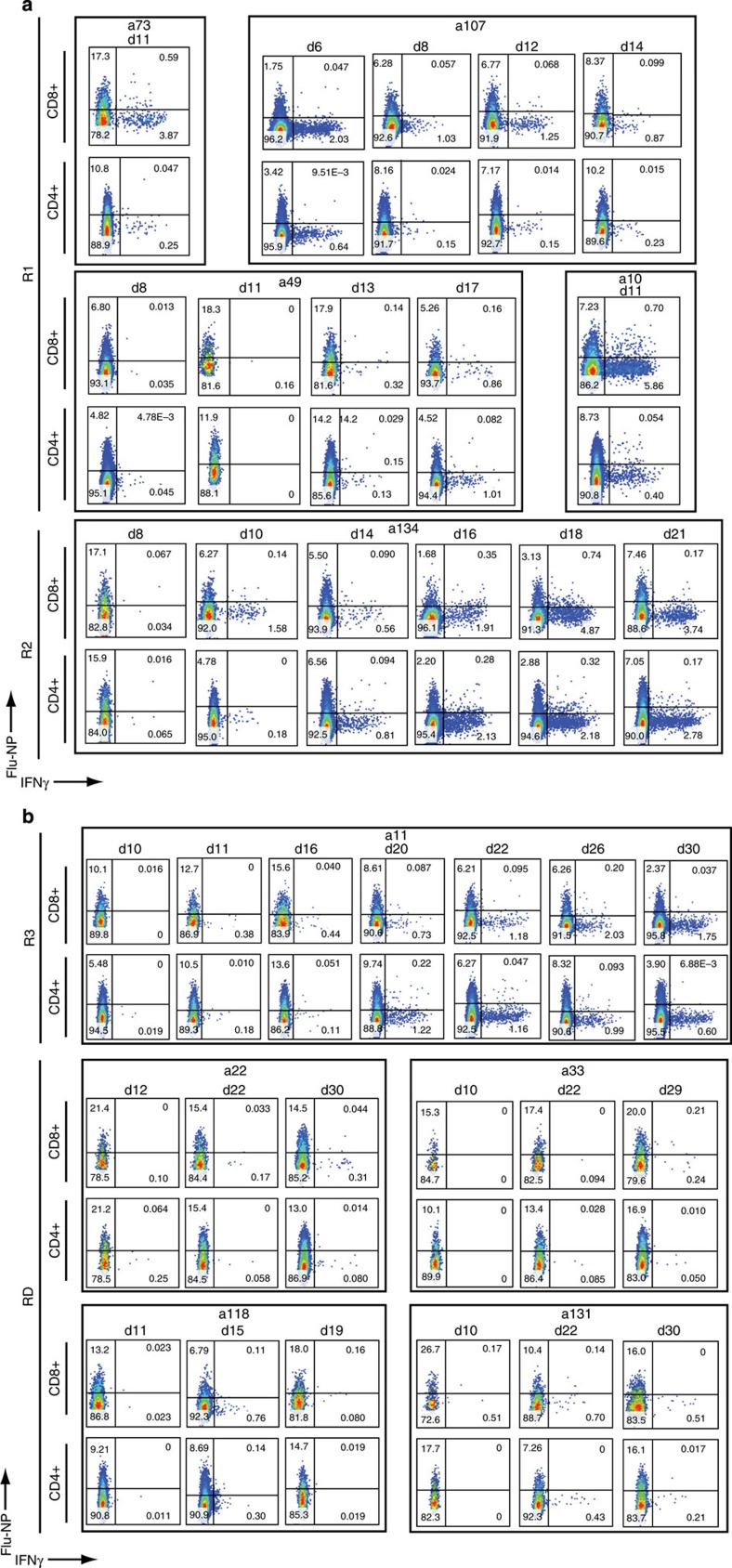
Kinetics of CD8^+^ and CD4^+^ T-cell responses in H7N9-infected patients. The frequency of H7N9-specific CD8^+^ (the upper row of each group) and CD4^+^ (the lower row of each group) T cells was determined by intracellular IFN-γ staining after stimulation of PBMCs with the A/Shanghai/H7N9/1 virus at MOI=4, at various time points after the onset of clinical symptoms. The patients were divided into three recovery groups, R1 (recovered d14–d18), R2 (recovered d21–d27), R3 (recovered d31–d35) and RD (the fatal outcome group), based on illness duration, hospital stay and disease outcome. H7N9 infection of CD8^+^ T cells was detected by the intracellular staining with the nucleoprotein-fluorescein isothiocyanate antibody to IAV-nucleoprotein.

**Figure 3:**
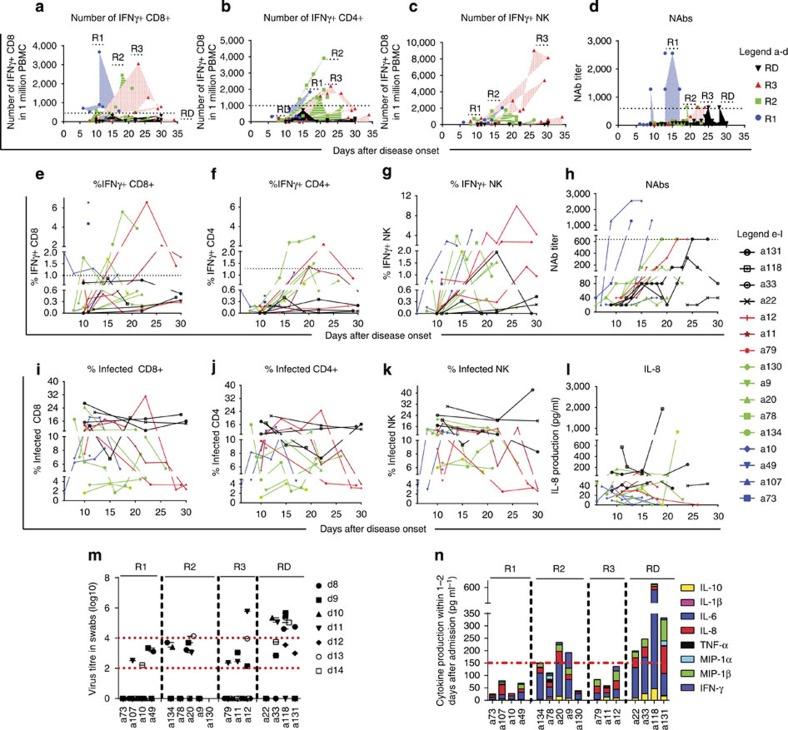
Multiparameter analysis of CD8^+^ T cells, CD4^+^ T cells, NK responses, antibody levels and the infection of cellular subsets in H7N9 patients. Kinetics of H7N9-specific CD8^+^, CD4^+^, NK and NAb responses are shown for (**a**–**d**) patient groups and (**e**–**i**) individual patients. Kinetics of IFN-γ-producing CD8^+^, CD4^+^ and NK responses are shown as (**a**–**c**) absolute numbers or (**e**–**g**) as frequencies, % IFN-γ-producing (**e**) CD8^+^ T cells of total CD8^+^ T cells, (**f**) CD4^+^ T cells of total CD4^+^ T cells and (**g**) NK cells of total NK cells. (**h**) Individual kinetics of NAbs during the disease course. (**i**–**k**) Infection level of CD8^+^, CD4^+^ and NK cells during the disease course detected by *in vitro* culture with H7N9 is shown. (**l**) Kinetics of plasma IL-8 during the disease course in individual patients, and the comparison of (**m**) viral load and (**n**) total serum cytokine with a disease outcome.

**Figure 4:**
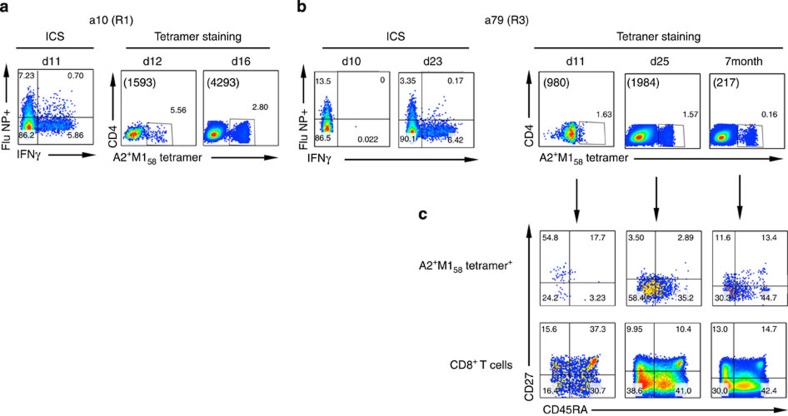
Phenotype of A*0201-M1_58_^+^-specific CD8^+^ T cells. PBMCs obtained from two HLA-A*0201^+^ patients (**a**) a10 (R1) and (**b**,**c**) a79 (R3) were stained with tetrameric complexes specific for the HLA-A*0201-M1_58_ epitope and a panel of mAbs against CD45RA, CD27, CD3, CD4 and CD8. (**a,b**) Comparable frequencies of influenza-specific CD8^+^ T-cell responses are shown by the H7N9-specific IFN-γ^-^based assay and the A*0201-M1_58_ tetramer staining. (**c**) The phenotype A*02:01-M1_58_^+^CD8^+^ T cells has been assessed on the basis of the expression of CD45RA and CD27. The absolute numbers of A*02:01-M1_58_^+^ CD8^+^ T cells are indicated in brackets (in per million PBMC). Cells were gated on dead cell marker^−^CD3^+^CD4^−^CD8^+^.

**Figure 5:**
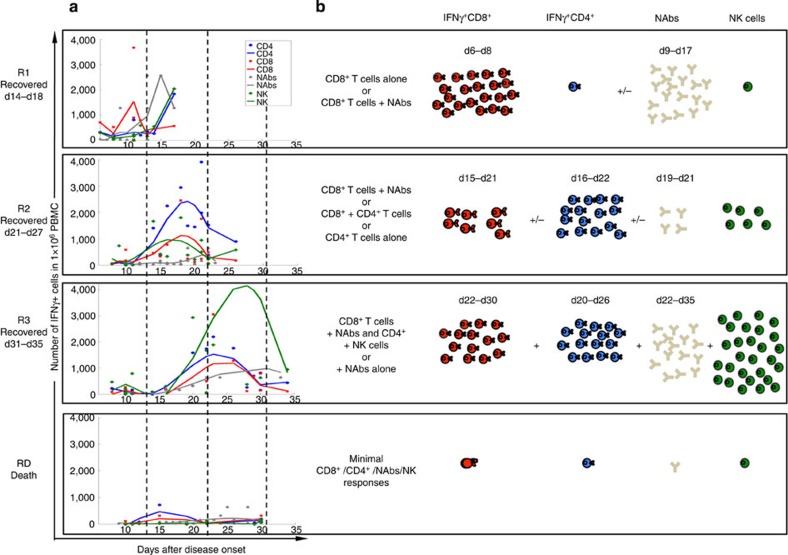
Proposed model of sequential recruitment of immune effectors during severe H7N9 disease. The absolute numbers of H7N9-specific IFN-γ-producing CD8^+^ T cells (red symbols), CD4^+^ T cells (blue symbols), NAbs (grey symbols) and NK cells (green symbols) are shown across different patient recovery (R1–R3) and fatality (RD) groups. (**a**) Least square polynomial regression was used to calculate and model the absolute numbers for H7N9-specific IFN-γ-producing CD8^+^ T cells, CD4^+^ T cells, antibodies NAbs and NK cells. The mean data are shown for each group and ‘No virus control’ values were subtracted. For NK cells in R3, three data points fall outside the scale (d26: value of 9,044; d29: value of 5,375; d30: value of 8,161). (**b**) Schematic representation of the immune cell sets important for the recovery from H7N9 across patient groups is shown.

Our results suggest that influenza-specific CD8^+^ T-cell responses occur early in patients who rapidly recovered from H7N9 disease. Analysis of the early-recovery R1 group (Figs 2 and 3a,e; Table 1; Figs 4a and 5) showed that, indicative of a protective effect, these IAV-specific CTLs were prominent in numbers around d10 (1.97% on d6 in a107; 4.37% on d11 in a73 and 6.55% in a10 on d11). The d11 findings for patient a10, for example, show CTL and T_H_ frequencies 6.55% and 0.45%, respectively, with NAb still being ≤40 on d18 (Figs 2 and 3a,e; Table 1; Fig. 5a). The remaining three R1 patients showed high NAb titres 2–3d after (a73 and a107), or concurrent with (a49; Table 1) high early CTL numbers, suggesting that these two components of immunity work together to promote protection. Overall, the 10–14d early CTL counts for R1 versus R2 and R3 (Fig. 3a) suggest that these patients benefited from rapid recall of heterosubtypic, cross-reactive CD8^+^ T-cell memory established by prior exposure to IAV subtypes that share immunogenic peptides (Fig. 5).

### Later recovery involves a network of cellular responses

In the R2 group (recovered between d21 and d27), prominent (>1%) CTL responses were detected only in patients a134 and a78 (Fig. 2 and Table 1), with peak counts being recorded much later (d19 and d10, for R2 versus R1 (Fig. 3e). Strikingly, the R2 T_H_ responses were much more robust, and appeared to be associated with recovery in patients a20, a9 and a130, in whom the CTL and NAb responses on d10 and d21 were minimal (Table 1 and Figs 2 and 3b,d–f,h). Peaking between d16 and d26 (Table 1 and Figs 2 and 3), it seems that H7N9-specific T_H_ set is elicited relatively late compared with (where available) the CTLs (Fig. 3ab). With the exception of a78, NAb levels in the R2 patients remained relatively low (Table 1 and Fig. 3). The ‘intermediate’ (by 4 weeks) recovery profile from severe H7N9 infection may thus reflect that protection is mediated via a delayed CTL response plus the later emergent T_H_ effectors.

The R3 patients recovering after >30d of hospitalization showed very low H7N9-specific CD8^+^ and CD4^+^ T-cell counts on ∼d10, which increased by ⩾d20 (Fig. 3a,b,d,f), in the presence of substantial NAb titres (Fig. 3d,h) and high NK cell numbers (Figs 3c,g and 5 and Supplementary Fig. 4). This profile was unique to R3, suggesting that antigen persistence drives the emergence and augmentation of a broader response spectrum than that associated with more rapid recovery (R2 versus R3, Fig. 3c,g and Supplementary Fig. 2). The magnitudes of the H7N9-specific CD4^+^ T cell and NAb responses did not seem to be linked to NAb titres for a134, a20, a9 and a130, remaining low (NAsb ≤640) until the time of discharge in the presence of significant (>1%) T_H_ counts (Figs 2 and 3h and Table 1) In addition, our finding that increased NK cell counts follow the CTL response (Fig. 3a,c,e,g) is consistent with *in vitro* experiments^18^, suggesting that NK activation depends on CD8^+^ T-cell involvement.

Although it is possible that we are not detecting high levels of H7N9-specific IFN-γ-producing NK cells in the R1 group, as the patients got released from the hospital, we have analysed H7N9-specific NK cell responses in patients from R2, R3 and RD groups at similar time points and found that only the R3 group was characterized by robust NK cell responses. When we compared groups R2, R3 and RD on ∼d22, we observed that, although %NK cells within the PBMCs and %IFN-γ^+^NK cells within the NK cell population were similar across the three groups, the frequency of IFN-γ-producing NK cells in PBMCs was significantly higher for the R3 subgroup compared with the RD subgroup (Supplementary Fig. 5).

Furthermore, to assess differences in IFN-γ-producing CD8^+^, CD4^+^ and NK cells between patients’ subgroups, we analysed immune responses divided by ‘time window’ reflecting patients’ recovery. The data showed statistically significant differences between the maximum number of IFN-γ-producing CD8^+^ T cells (for R1), CD4^+^ (for R2) and NK cells (for R3) against the fatal RD group at the time of the subgroup recovery (Supplementary Fig. 6). Thus, when we subgroup the patients, the results support our proposed model of consequential recruitment of immune effectors for recovery from severe H7N9 influenza disease, with the strongest CD8^+^ T-cell responses for the R1 group, strongest CD4^+^ T-cell responses for the R2 group and strongest NK cell responses for the R3 group. Thus, IFN-γ-producing CD8^+^ T cells in R1, CD4^+^ T cells in R2 and NK cells in R3 ‘survival’ groups were markedly (and significantly) increased when compared with patients who died following H7N9 infection, suggesting that different immune responses at different times might be associated with recovery when compared with patients who died from H7N9 disease.

Co-expression of the surface TCRα7.2, CD161 and CD8α markers is characteristic of most MAIT cells; however, as CD161 was downregulated in our patients, we defined this population as CD8^+^TCRα7.2^+^. Analyses of CD8^+^TCRα7.2^+^ and γδ subsets showed no obvious divergence in overall frequencies (Supplementary Figs 7 and 8), although the CD8^+^TCRα7.2^+^ T-cell counts for R1 were significantly greater (*P*<0.01) than those for R2, R3, RD and elderly uninfected controls. Some patients (a11 d20, a11 d26, a107 d6, a107 d8, a107 d14, a118 d15, a134 d16 and a134 d18) had IFN-γ-producing MAIT cells at frequencies of 1–5%, and IFNγ-producing γδ T cells (1–4%) were found regardless of the patient group (including the RD group). There is thus some indications that innate γδ T cells and MAIT cells may play a part in immunity to the H7N9 IAV, although their role remains to be dissected.

Our data suggest that in the absence of substantial pre-existing, H7N9-specific CD8^+^ T-cell memory (R1 group), the recruitment of naive (or low-frequency memory) CTLs, together with T_H_ cells, NAbs and NK effectors, provides a protective mechanism that becomes more obvious with the persistence of severe disease in those who will eventually recover (Fig. 5). This also suggests that designing new influenza vaccines that establish robust and numerically sizable CD8^+^ T-cell memory pools can provide significant protections against novel, virulent IAV infections.

### Lack of cellular and humoral responses in fatal cases

In contrast to the recovery groups R1–R3, the RD patients showed minimal CTL, T_H_ and NK responses on d10, d21 and d30 and, with the exception of a131, with evidence of NAb production on d25 (Figs 2, 3, 5 and Table 1). Thus, it appears that in the absence of cellular responses, the delayed NAb antibody responses may be insufficient for survival from the severe H7N9 influenza disease, although may extend the hospitalization time. There were no significant differences in the age of the patients between the ‘survival’ and ‘death’ groups or across the subgroups. Furthermore, there was no correlation between the age of the donors and H7N9-specific CTLs (Supplementary Fig. 1). Patients from R1, R2 and R3 but not RD could mount CD8^+^ T-cell responses irrespectively of the age. Whether this reflects a lack of appropriate precursor cells, or immunosuppression caused by early, severe inflammation (Fig. 3l,n) is far from clear. Significant correlations were, however, observed for both IAV-specific CD4^+^ and CD8^+^ T-cell numbers versus lymphocyte counts (Supplementary Fig. 9). Furthermore, all patients who recovered had virus-specific CD8^+^/CD4^+^ (IFN-**γ**^**+**^CD8^+^ >400 per million PBMC and/or IFN-**γ**^+^CD4^+^ 800 per million PBMC) at least at one time point after infection. Low frequencies of CD4^+^/CD8^+^ T cells were previously observed in the peripheral blood of H7N9 patients, especially those with fatal infections^5^. This could reflect the prolonged accumulation of IAV-specific T cells at the site of infection. However, the magnitude of IAV-specific T cells in blood PBMCs represents active migration of IAV-specific T cells between lymph nodes and infection sites, and correlate with the magnitude of IAV-specific T-cell responses in the lung and in the lymph node^19^. Even so, it seems that checking early on for serum cytokines and responder CD8^+^ and CD4^+^ T cells may be a good predictor of outcomes and suggest alternative treatment strategies.

### Infection rates of CD8^+^, CD4^+^ T cells and NK cells

We also found that the incidence of IAV-infected CD8^+^ and CD4^+^ T cells (in our *in vitro* assay) changes during the course of severe, non-fatal H7N9 disease, decreasing from >10% early on to 2–4% in the recovery phase. Correlating with the lack of effective immunity, the high *in vitro* CD4^+^/CD8^+^/NK cell infection rates (10.4–26.8%) persisted for those who succumbed (Fig. 3i–k), while the infection rate fall for CD8^+^ T cells (d18 for R1, d14 and d22 for R2 and d22 and d30 for R3) was directly related to recovery (Fig. 3i) and inversely correlated with H7N9-specific CTL and T_H_ frequencies. Unexpected results are that the infection rate changes as the disease progresses even in CD8^+^/CD4^+^/NK cells from the same H7N9 patients, when the assay was performed on the same day. This may indicate that (1) the inflammation at the early stages of disease may influence the infection sensitivity of CD8^+^ T cells to H7N9 virus; (2) immune cells could be resistant to H7N9 infection in the late recovery phase; or (3) the H7N9-infection-sensitive cells died or were selected out during the illness. The latter could explain, at least in part, the lower CD8^+^ and CD4^+^ T-cell numbers in patients who died from H7N9 infection. Further analyses of the part played by the inflammatory/cytokine milieu and/or cell-surface phenotype changes (receptor/co-receptor expression) may prove instructive.

### A*0201^+^M1_58_^+^ CTLs in the symptomatic and recovery phases

As the response to the HLA-A*02:01-restricted M1_58_ epitope contributes significantly to the overall virus-specific CTL response^20^, we have verified our observations in epitope-specific populations. The CTLs from two (out of four; Supplementary Table 1) HLA-A*02:01^+^ patients (a10 and a79) were stained with an A*02:01-^+^M1_58_ tetramer, and monoclonal antibodies (mAbs) to CD45RA and CD27 (Fig. 4ab), to distinguish between the naive (CD27^+^ and CD45RA^+^), effector memory (TEM, CD27^−^/CD45RA^−^), terminally differentiated EM (TEMRA, CD45RA^+^/CD27^−^) and central memory (TCM, CD27^+^/CD45RA^−^) T cell subsets (Fig. 4c, lower panel). The response magnitude for the tetramer^+^CD8^+^ CTLs recovered from a10 on d12 (5.56%) was comparable to that found by the H7N9-specific IFN-γ^+^ ICS assay on d11 (6.56%), indicating the dominance of the A*02:01-M1_58_-specific subsets. Moreover, although the frequency of A*02:01-M1_58_^+^CD8^+^ T cells decreased to 2.8% by d16, the absolute numbers increased from 1,593 to 4,293 per million PBMC (Fig. 4a).

For patient a79, the frequency of A*02:01-M1_58_^+^CD8^+^ CTLs was comparable on d11 and d25 (1.63 and 1.57%), while the number increased from 980 to 1,984 per million PBMCs (Fig. 4b). These epitope-specific cells changed phenotypically (Fig. 4c), with the majority being initially CD45RA^−^ (either CD27^+^ or CD27^−^). The TEMRA phenotype (CD45RA^+^CD27^−^) consistent with virus clearance emerged by d25. Although the frequency of the A*0201-M1_58_^+^CD8^+^ CTLs was markedly reduced to 0.16% (217 per million PBMCs) by 7 months after disease onset, the phenotype then was comparable to that observed on d25, with effector and TEMRA cells constituting the majority of the tetramer^+^CD8^+^ sets (Fig. 4c). Furthermore, the observation that the d11 A*0201-M1_58_^+^CD8^+^ T cells (Fig. 4c) displayed a TCM or TEM phenotype (substantially CD27^−^) suggests that they originated from an established memory pool.

### Transcriptomic analysis

**Figure 6:**
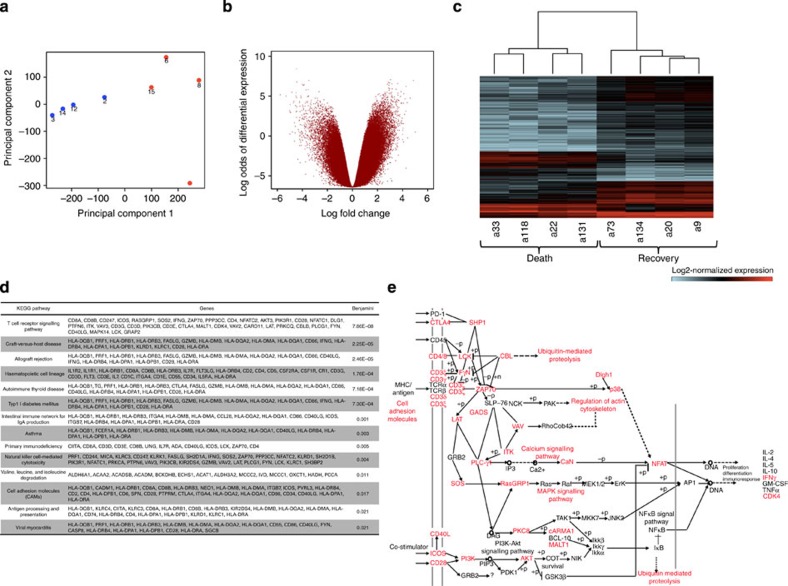
Transcriptomic analysis confirms increased cellular activation in the patient recovery group. (**a**) Principal components analysis shows clear global transcriptional differences between the recovery (blue) and fatal (red) patient groups along PC1. (**b**) A volcano plot showing substantial differential gene probe set expression between recovery and fatal patient groups with similar numbers of probes up- or downregulated. (**c**) A heatmap showing the diversity of expression patterns for hierarchically clustered gene probes (rows; BH-adjusted *P* value<0.05 and log fold change>2.0) versus patients (columns) from the fatal and recovery groups. (**d**) Significantly enriched KEGG pathways among the differentially expressed probes, together with their corresponding gene annotations. (**e**) A schematic of the most significantly enriched KEGG pathway for TCR signalling, with genes harbouring at least one differentially expressed probe marked with red.

To identify molecular signatures correlated with H7N9 disease outcomes, we analysed the immune-related transcriptomes for PBMCs from four of the recovered (a73 d11, a134 d22, a20 d22 and a9 d21) and four of the fatal cases (a22 d22, a33 d21, a118 d19 and a131 d22). Total RNA was run on Affymetrix Human Gene ST 2.0 arrays and details of the probe set-level normalization and differential expression analysis are given in the Methods. Principal component analysis of the log2-normalized gene expression values showed clear, global differences between the transcriptomes in fatal versus recovery outcomes, to the extent that the two groups could be linearly discriminated along the first principal component (Fig. 6a). Differential expression analysis established that there was a substantial excess of differentially expressed probes between the two groups (Fig. 6b) with a diversity of expression patterns (Fig. 6c). In total, 4,835 probe sets on the array corresponding to 2,055 genes showed differential expression at a Benjamini–Hochberg (BH)-adjusted *P* value<0.05 and a log2 fold-change of ⩾2.0. KEGG pathway analysis (Fig. 6d,e) showed a significant enrichment for genes in various immune-related pathways with by far the strongest enrichment in T-cell receptor (TCR) signalling (BH-adjusted *P* value=7 × 10^−8^). Of the 165 probe sets corresponding to genes involved in TCR signalling, 159 (96%) were upregulated >4 fold in the recovery group, indicating that T-cell activation at a transcriptional level is a predictor of H7N9 disease outcome.

## Discussion

Our study utilized a unique cohort from the first wave of A/H7N9 outbreak in 2013. To the best of our knowledge, these samples (*n*=16) represent the only longitudinal PBMC samples from the first wave of an H7N9 outbreak. We analysed patients’ PBMCs from different time points to maximize our sample size. Using this cohort, we present the first dissection of immunity during the course of human influenza, focusing on the effector mechanisms that promote recovery from severe H7N9 disease. The most striking observation is that patients with a prominent, early H7N9-specific CD8^+^ CTL response recovered faster than those who lacked what seemed to be pre-existing, heterosubtypic CTL memory. In the absence of such early CTL recall, those hospitalized patients who recovered later established a more complex profile of immune effector mechanisms before disease resolution. The longer the hospitalization with severe H7N9 disease, the more diverse these immune responses became, involving also CD4^+^ T_H_ cells, and NAbs then, in those who recovered late, the emergence of prominent, innate NK cell populations. Indeed, the severity and duration of H7N9-induced pathology and respiratory compromise allowed us to access the emergence of a much more complex pattern that has been seen in the past for readily resolved human cases caused by less pathogenic IAVs, and for the milder strains used in mouse experiments^11^,^12^,^17^,^19^,^21^,^22^.

We acknowledge that our study is based on the longitudinal samples obtained from 16 individual patients, with diversity of clinical courses, comorbidities and the resultant cellular and humoral immune responses to H7N9. However, despite this, we believe that our study provides the first suggestion that a diversity of immune mechanisms is associated with human influenza disease length and outcome. Further studies are needed to fully understand the mechanisms driving the recovery from influenza virus infection across different disease severities, from asymptomatic through mild to severe influenza disease in a large patient cohort. Our data, however, provide strong arguments for a thorough dissection of several immune responses in a human disease rather than a single cellular response, such as CD8^+^ or CD4^+^ T cells *per se*, as the results on the importance of such single immune response may differ depending on the nature of other immune responses.

Given that prominent, H7N9-specific CD8^+^ CTLs could be found as early as d6 after disease onset emphasizes the likelihood of recall from robust, cross-reactive CD8^+^ memory T-cell pools established by prior infection with other IAVs that share immunogenic peptides. Recent findings from our^23^ and the Rimmelzwaan^24^ group established that a substantial proportion of peptide+ class I major histocompatibility complex (MHC) glycoprotein (pMHCI) epitope-specific CD8^+^ memory T cells induced by previous epidemic or pandemic IAV exposure will be cross-reactive for H7N9. Furthermore, the HLA-I alleles capable of presenting conserved IAV peptides (HLA-2 is especially prominent^20^) vary greatly across different ethnicities^23^, explaining why some groups that are known to be susceptible to influenza are at substantially greater risk. While the estimated coverage for such IAV-specific, pre-existing CTL-mediated immunity to H7N9 within Caucasians is 57%, the frequency is lower for Oriental population (37%) and markedly reduced in Indigenous Alaskans and Australians (16%; ref. 23).

All the H7N9 cases we analysed in our present study were ethnic Chinese, where we would expect at least 30% to have pre-existing CTL memory pools that could be rapidly recalled by H7N9 infection. How effective this protective mechanism might be would, of course, be influenced by a variety of factors, including the age and lapse of time since an individual’s latest IAV experience^25^,^26^. Although functional IAV-specific CTL memory is maintained for the life of a laboratory mouse^22^,^27^, the durability of such populations in humans, especially in the elderly, is understudied. A 1977–1982 analysis by McMichael *et al*.^28^ showed contraction of T-cell memory using PBMCs in the then-standard ^51^Cr-release assay. It is, however, possible that diminished target cell lysis could be partially related to the loss of the CTL-killing capacity^29^, or reflect diminished cytolytic molecule expression (gzm A and gzm B) in the long-term memory CD8^+^ sets^30^. Elegant analyses of memory CTL persistence for vaccinia virus suggest that the estimated half-life for the CD8^+^ set is between 8 and 15 years^31^,^32^, with ∼50% of individuals showing substantial loss within 20 years of immunization^31^. Further analysis using a spectrum of IAV peptide+HLA tetramers are merited to pursue the question of CTL persistence, especially in the elderly, so that we could develop a clearer view of the utility of IAV vaccines designed to boost such cross-reactive (against novel pandemic or outbreak strains) immunity.

Two recent studies looked closely at the IAV-specific CD8^+^ CTLs^12^ and the CD4^+^ T_H_ population^11^ in human A/H1N1pdm09 and A/H3N2 infections. While thorough dissection of such immune responses during this H7N9 outbreak indicated that both CD8^+^ and CD4^+^ T cells are important, the results reported here additionally highlight the fact that the prominence of one or other potential effector mechanisms will vary depending on the duration of severe disease and the timing relative to recovery. Such kinetic variation in relative response prevalence no doubt explains why higher levels of CD4^+^ T_H_ cells versus CD8^+^ CTLs were found previously for PBMCs from patients with severe A/H1N1pdm09 disease^33^. That describes exactly what we found here for the situation where the onset of CTL-mediated immunity was delayed, leading to a more prominent T_H_ response.

Thus, our present, thorough kinetic analysis that relates the prevalence of various effector populations and mechanisms to disease severity and time to recovery provides new insights into the progression of IAV-induced disease. The findings indicate how, using rapid PCR to monitor (for example) TCR signalling in PBMCs, we might predict whether or not the disease will progress to a fatal conclusion, providing a prognostic feature that might suggest the use of new antiviral drugs or, perhaps, the use of mAbs that (using contemporary technology) could be developed very rapidly against a recently emerged outbreak, seasonal epidemic or pandemic IAV. The results also have implications for novel immunogen design and deployment, based on the central finding that cross-reactive CTL memory pools generated following encounters with seasonal or pandemic IAVs (or potentially by a CTL-directed vaccine) can limit the severity of an emerging, and perhaps very severe and widespread, disease event.

## Methods

### Patient cohort

Sixteen H7N9 patients, confirmed using qPCR, were admitted to the Shanghai Public Health Clinical Center (SHAPHC) between March and August 2013 (ref. 4). All of the H7N9-infected patients reported a fever >38.7 °C and cough. Twelve patients recovered between d14 and d35 after the disease onset, while the remainder died on d19, d64, d70 and d76. The clinical information included patient demographics, daily monitoring of treatment, disease progression and recovery. Virus titres were determined using qPCR for cDNA copies from throat swabs as log_10_ per ml of viral transport medium. Informed consent was obtained from all participants. The overall study was reviewed and approved by the SHAPHC Ethics Committee. The disease course was described as a number of days after a disease onset.

### PBMC isolation and pseudovirus-based neutralization assay

PBMCs were prepared from 3 to 10 ml of patients’ peripheral blood using Ficoll-plaque density gradient centrifugation. For immune studies, the A/Shanghai/01/2013 (H7N9) virus^1^ was propagated in MDCK cells and titres were expressed as TCID_50_ in MDCK cells. Microneutralizing antibodies were detected with a luciferase reporter-based NAb assay, using a nonreplicative human immunodeficiency virus backbone carrying influenza A H7 and N9 (ref. 14). The neutralizing titre of human sera was defined as the highest serum dilution that gave ⩾80% inhibitory concentration (IC_80_) of the luciferase signal in virus-infected MDCK cells. On the basis of a previous study^14^, we had defined the IC_80_ and an antibody titre of 1:40 as the best discriminators between H7N9-infected patients and noninfected controls. As previously published^12^, an NAb titre of ⩾640 was used to define a recovery cutoff.

### *Ex vivo* H7N9 influenza fluorescence-activated cell sorting-based cellular immunity assay

The immune assays were performed in a PC3 laboratory as blinded experiments with respect to the clinical data. Briefly, 1.5 million patient PBMCs were stimulated with live A/Shanghai/01/2013 (H7N9) virus at MOI=4 for 1 h. Subsequently, 10% of FCS was added to the cultures and incubated at 37 °C and 5% CO_2_ for 3 h. Golgi Plug was added (BD, 1:2,000), and the cells were cultured under the same physical parameters for the next 18 h. As a negative control, PBMCs were exposed to RPMI medium containing 10% fetal calf serum (FCS) and penicillin/streptomycin (Sigma-Aldrich, USA). PBMCs were stained with dead cell discrimination marker (Live Dead Aqua, Life Technologies) in PBS for 15 min at room temperature, and then with a panel of surface mAbs in FACS buffer (2 mM EDTA, 0.5% bovine serum albumin) for 30 min on ice, including PECF594-CD3 (BD 562280, 1:200, clone UCHT1), PerCP-Cy5.5-CD8 (BD 341051, 1:50, clone SK1), BV605-CD161 (Biolegend 339915, 1:50, clone HP-3G10), APC (antigen-presenting cell)-H7-CD14 (BD 560180, 1:100, clone MφP9), APC-H7-CD19 (BD 560177, 1:100, clone SJ25C1), BV421-γδTCR (Biolegend 331218, 1:50, clone B1), PE (phycoerythrin)-TCR Vα7.2 (Biolegend 351706, 1:400, clone 3C10), BV650-CD4 (Biolegend 317435, 1:200, clone OKT4) and PE-Cy7-CD56 (BD 335791, 1:100, clone NCAM16.2). Subsequently, cells were fixed with BD fix/perm buffer on ice for 20 min, and then stained with a panel of intracellular markers in a BD Perm/wash buffer for 30 min on ice. The mAbs included AF700-IFN-γ (BD 557995, 1:200, clone B27), APC-tumour-necrosis factor-α (BD 340534, 1:50, clone 6401.1111) and fluorescein isothiocyanate-Flu Nucleoprotein (Gene-Tex 1:200, clone 1331). After two washes, cells were resuspended in 1% paraformaldehyde (PFA) FACS wash buffer for flow cytometry (BD LSR Fortessa). This newly established assay allowed us to dissect the role of CD8^+^ T cell, CD4^+^ T cell, NK cell, γδ T cells and MAIT cells during the course of severe H7N9 influenza infection. Samples were analysed with FlowJo software Version 10 (TreeStar).

### Statistical analyses

Least square polynomial regressions were used for the modelling of virus-specific CD8^+^ T cells, CD4^+^ T cells, NAbs and NK cells (Fig. 5). The comparison of highest numbers of CD8^+^, CD4^+^, NK cells and NAbs from each patient between survival and fatal groups was performed using Mann–Whitney test. For the subgroup analysis, a Kruskal–Wallis test was performed when three or more groups were compared, with Dunn’s post-tests on R1 (where present) and RD compared with all other groups. When two groups had data (after d27), Mann–Whitney test was used.

### Microarray-based peripheral blood transcriptome profiling

Peripheral blood (2 ml) was collected from each patient, preserved in PAXgene Blood RNA Tubes (QIAGEN, cat. no. 762125) and stored at −80 °C until use. Total RNA was extracted by using PAXgene Blood RNA Kit (QIAGEN, cat. no. 762134). Microarray-based transcriptome assay was performed by the Gene Tech (Shanghai) company using Affymetrix GeneChip Human Gene 2.0 ST Array (cat no. 902112). All analyses used the R statistical programming environment (v3.03). Raw CEL intensity files underwent ‘Perfect Match’ probe set-level normalization including background correction using robust multiarray averaging (RMA), quantile normalization and summarization of gene expression using the median published algorithm^34^. Probe set intensity distributions between arrays (before and after normalization) and plots of the log intensity ratio versus the average log intensity (MA plots) were assessed to determine whether any arrays showed excessive technical variability; all arrays passed inspection. Unexpressed probe sets, those which did not have significantly more fluorescence than background probes, were identified as those with a PSDABG *P* value>0.05; these were removed from subsequent analyses. The LIMMA package was utilized to identify differentially expressed probe sets^35^. The death and recovery sets were compared using an empirical Bayes procedure to moderate s.e., and a *t*-test was used to test for difference in expression levels. Corresponding *P* values were adjusted for multiple testing using the BH approach. Differentially expressed genes were defined by adjusted *P* value <0.05 and an absolute log_2_ fold-change ⩾2.0, and KEGG Pathway analysis was performed using the NIAID DAVID tool (v6.7) (ref. 36), where multiple probe sets corresponding to a single gene were collapsed into a single entry. Significant functional enrichment was determined using a modified Fisher exact test (EASE score) and a BH adjusted *P* value of <0.05.

## Additional information

**Accession codes:** The microarray data have been deposited in the ArrayExpress database with accession code E-MTAB-3005.

**How to cite this article:** Wang, Z. *et al*. Recovery from severe H7N9 disease is associated with diverse response mechanisms dominated by CD8^+^ T cells. *Nat. Commun.* 6:6833 doi: 10.1038/ncomms7833 (2015).

## Supplementary Material

Supplementary Information Supplementary Figures 1-9 and Supplementary Table 1

## Figures and Tables

**Table 1:**
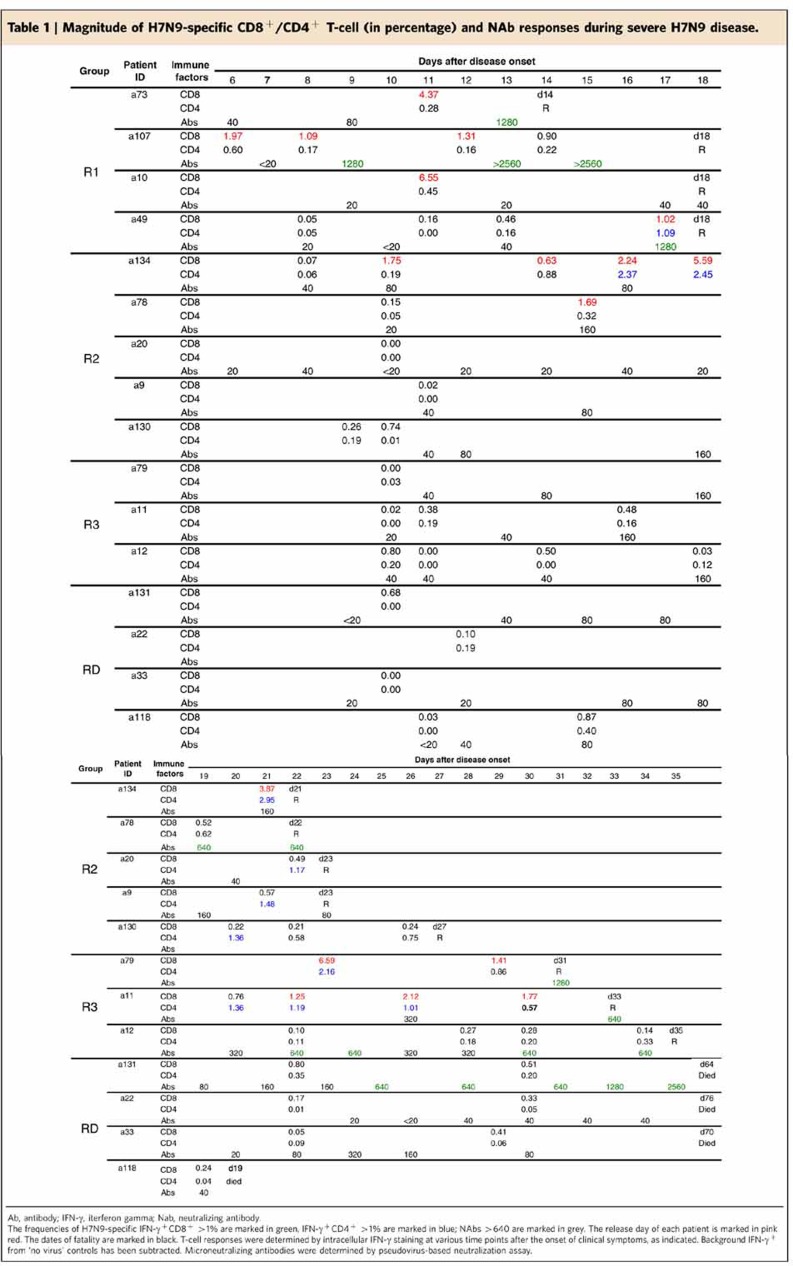
Magnitude of H7N9-specific CD8^+^/CD4^+^ T-cell (in percentage) and NAb responses during severe H7N9 disease.
