# Supplementary material for: Recovery from severe H7N9 disease is associated with diverse response mechanisms dominated by CD8+ T cells
Source: Nat Commun. 2015 May 13;6:6833. doi: 10.1038/ncomms7833 (PMC4479016; doi:10.1038/ncomms7833)
Supplement: Supplementary Information — Supplementary Figures 1-9 and Supplementary Table 1 [file ncomms7833-s1.pdf]

# SUPPLEMENTARY FIGURES

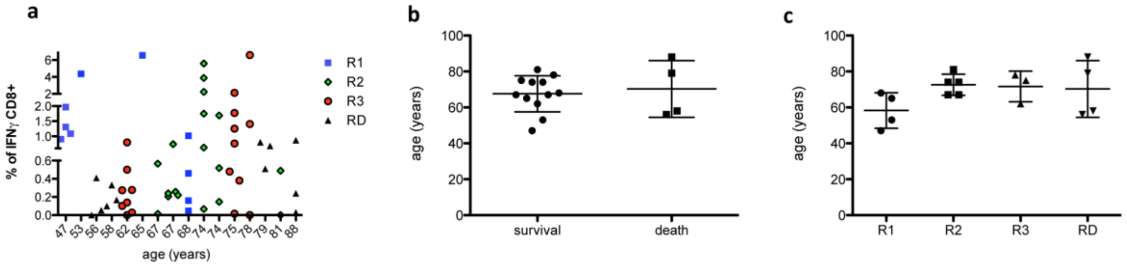


Supplementary Figure S1 Wang *et al*

**Supplementary Fig.1. Lack of correlation between the age of H7N9-infected patients and the CTL response, disease outcome or disease length.** **(a)** CTL response distribution across patients' age; **(b)** age distribution for the survival and death patients' groups, and **(c)** between different subgroups (R1, R2, R3 and RD).

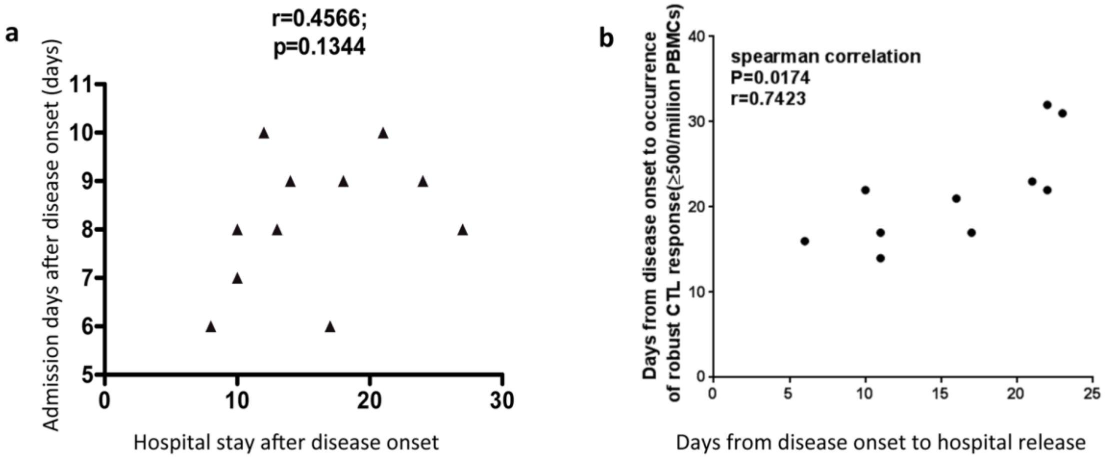


Supplementary Figure 2 Wang et al

**Supplementary Fig.2. Relationships between (a) admission days after disease onset and the length of hospital stay; and (b) the emergence of effective virus-specific CD8$^{+}$ T cells and the disease length.** **(a)** No significant difference between the admission days after disease onset and the length of hospital stay was found, as analyzed by non-parametric correlation analysis (Spearman). **(b)** Days from disease onset to emergence of effective virus-specific CD8$^{+}$ T cells strongly correlate with the disease length. The length of hospital stay was not affected by the admission day after disease onset. The analysis was performed by a non-parametric correlation Spearman test.

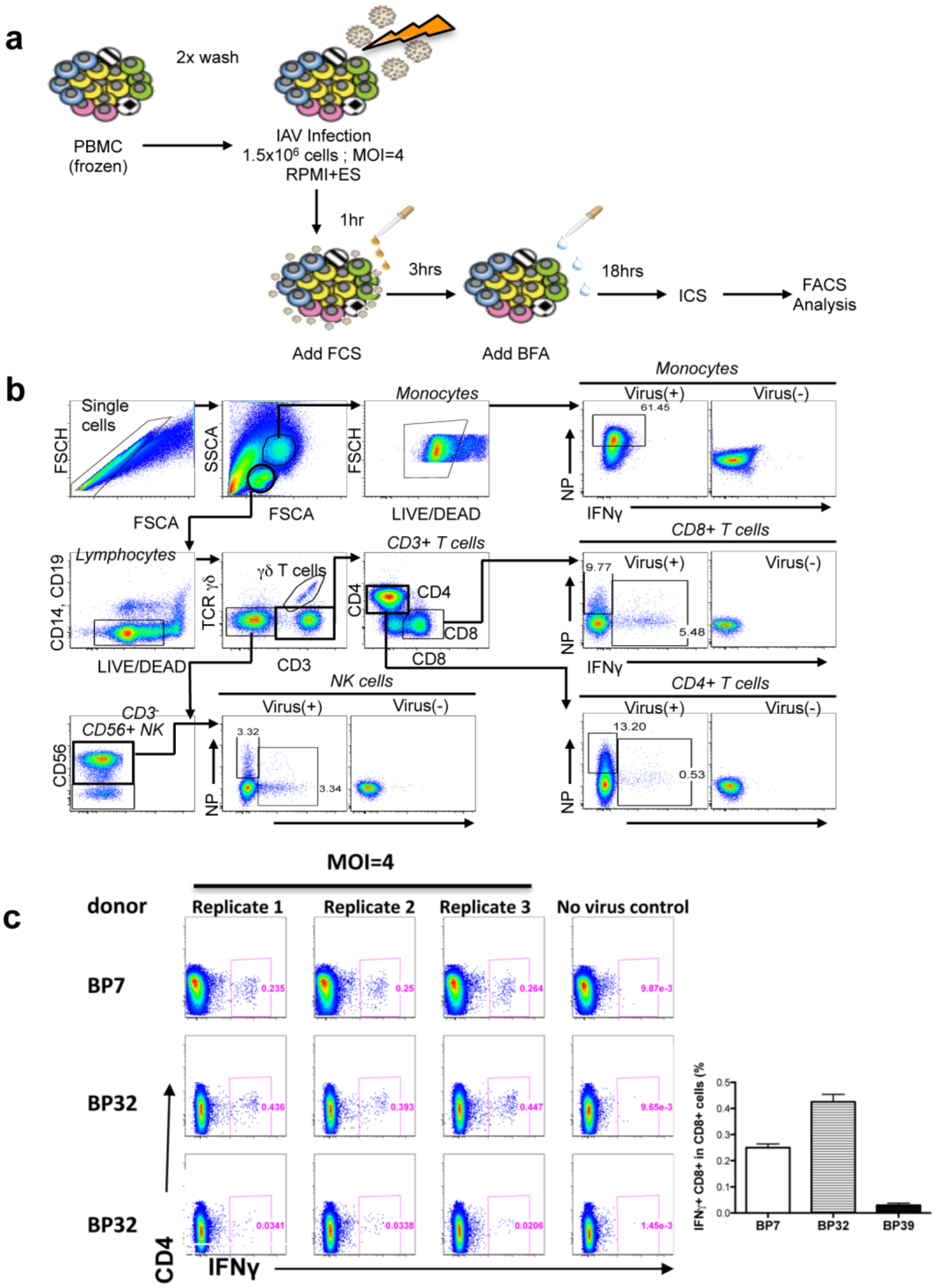


Supplementary Figure S3 Wang *et al*

**Supplementary Fig. 3. Simultaneous analysis of innate and adaptive immunity towards influenza H7N9 virus in patients hospitalized with severe H7N9 disease. (a)** Patient PBMCs (1x10$^{6}$) were stimulated with live A/Shanghai/01/2013 (H7N9) virus at MOI=4 for 1h, followed by addition of 10% of FCS and Golgi Plug. As a negative control, PBMCs were stimulated with RPMI medium containing 10% FCS. PBMCs were

stained for Live-dead Aqua 525 and a panel of antibodies: PECF594-CD3, Percp-cy5.5-CD8, BV605-CD161, APC-H7-CD14, APC-H7-CD19, BV421-γδTCR, PE- TCR vα7.2, BV650-CD4, PE-Cy7-CD56. Cells were fixed and stained with intracellular antibodies AF700-IFNγ, APC-TNFα and FITC-Flu NP. After 2 washes, cells were analysed by flow cytometry (BD LSR Fortessa). The immune assays were performed in a PC3 laboratory as blinded experiments. (b) Gating of $CD8^+$, $CD4^+$, NK, γδ and monocytes is shown**; (c)** The reproducibility of the assay was verified across different MOIs (multiplicity of infection; data not shown) and using different replicates at MOI of 4.

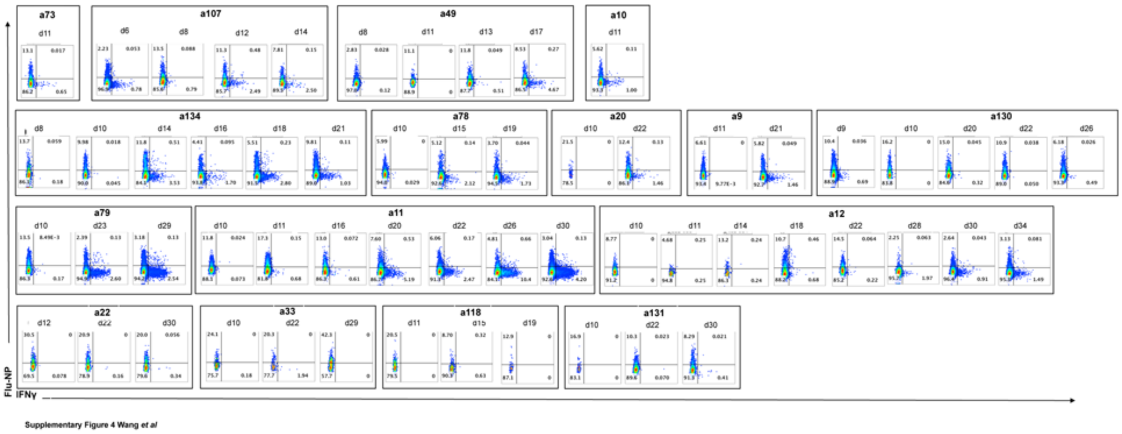


**Supplementary Fig. 4. Kinetics of IFN-γ producing NK cells.** The frequency of H7N9-specific NK cells producing IFN-γ after stimulation of PBMCs with A/Shanghai/H7N9/1 virus at MOI=4 was assessed by an Intracellular Cytokine Staining (ICS), as described in **Fig.S3**. The patients were divided into three recovery groups, R1 (recovered d14-d18), R2 (recovered d21-27), R3 (recovered d31-35) and RD (the fatal outcome group) based on the illness duration, the hospital stay and the disease outcome. Cells were gated on $CD56^+CD3^-$ population. H7N9-infected NK cells were detected by intracellular staining with the NP-FITC antibody.

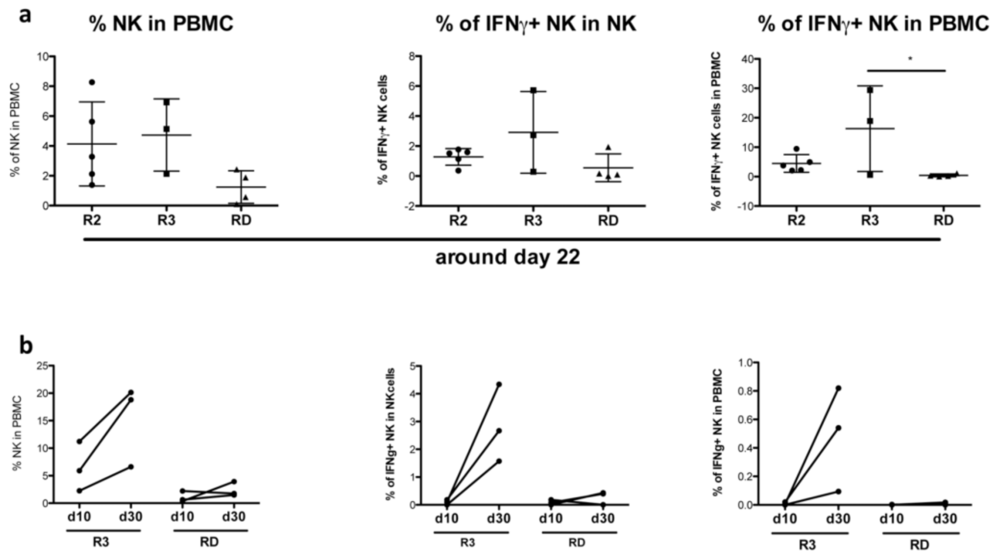


Supplementary Figure S5 Wang *et al*

**Supplementary Fig. 5. Elevated H7N9-specific NK response in the R3 group at d22 to d30.** **(a)** Increased frequency of IFNγ$^+$ NK cells was observed between R3 and RD groups in PBMCs but not IFNγ$^+$NK cells in NK cells or %NK cells in PBMCs. **(b)** The increase from d10 to d30 in NK frequency in PBMC, IFNγ$^+$NK frequency in NK cells and IFNγ$^+$NK frequency in PBMCs were only observed in R3, but not in RD.

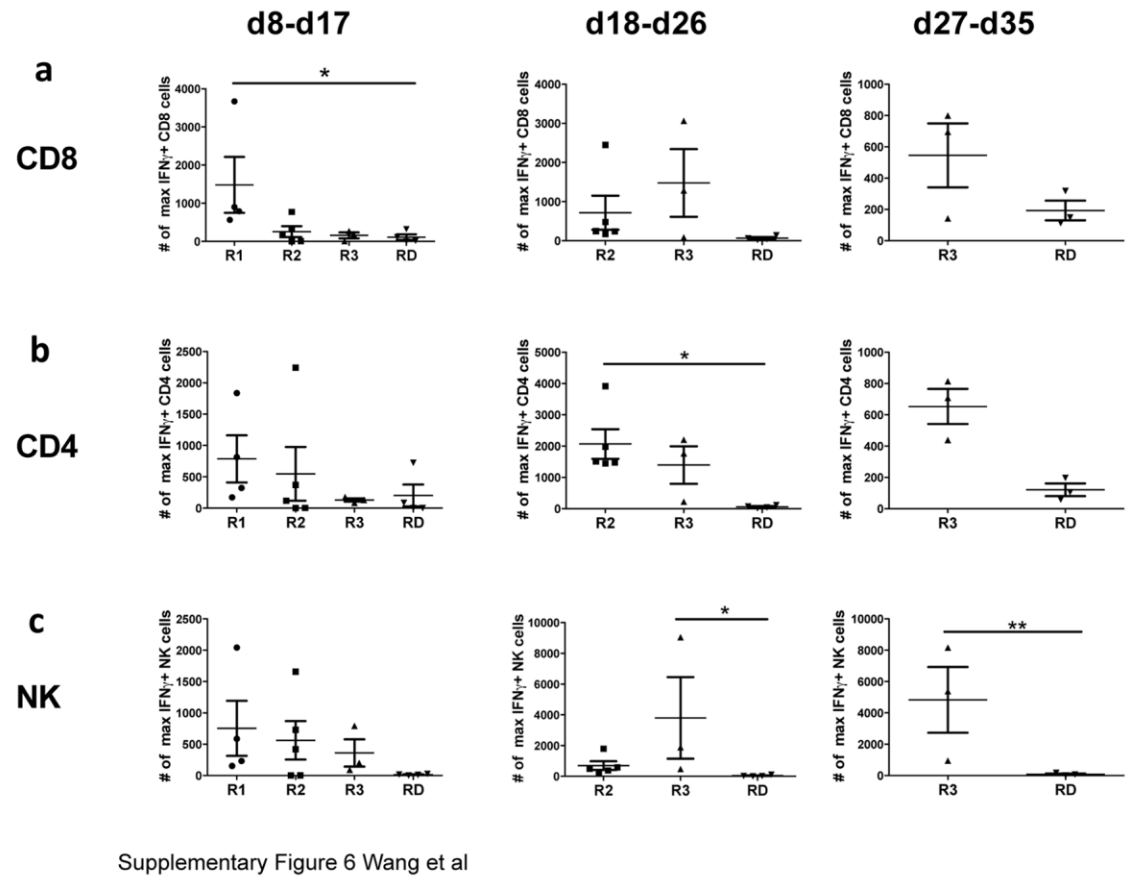


**Supplementary Fig.6. Elevated IFN-γ-producing CD8$^+$, CD4$^+$ and NK cell responses correlate with the H7N9 disease recovery at different time periods in different subgroups.** H7N9-specific IFN-γ producing **(a)** CD8$^+$ T cells in R1 during d8-d17; **(b)** CD4$^+$ T cells in R2 during d18-d26; **(c)** NK cells in R3 during d18-d35 were significantly increased compared to the RD patient group, as detected by IFN-γ ICS following 18 hrs stimulations with A/H7N9 virus. The comparison of the highest numbers of CD8$^+$, CD4$^+$, NK cells and NAbs during different periods of each patient between subgroups was performed using *K-wallis* test.

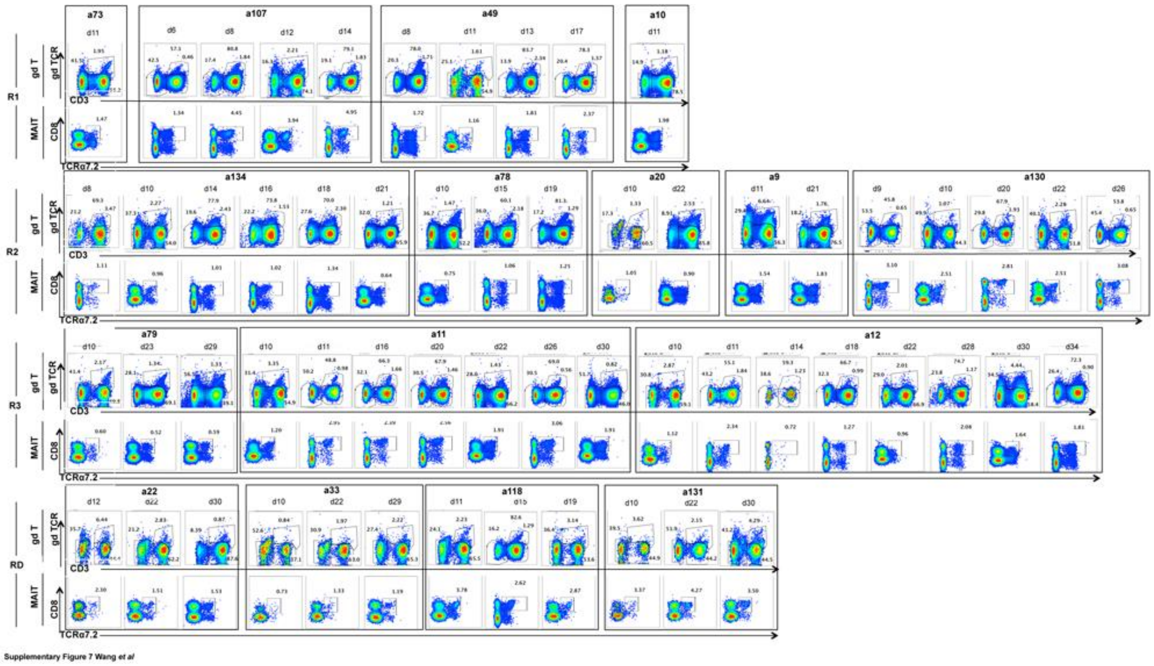


**Supplementary Fig. 7. The frequency of MAIT and δ T cells in H7N9-infected patients during influenza disease.** The frequency of MAIT (the upper row for each group) and δ T (the lower row for each group) cells was measured after stimulation of PBMCs with A/Shanghai/H7N9/1 virus at various time points after the onset of clinical symptoms. Cells were gated on death-marker$^-$CD3$^+$CD8$^+$TCRα7.2$^+$ and death-marker$^-$CD3$^+$TCRγδ $^+$ populations.

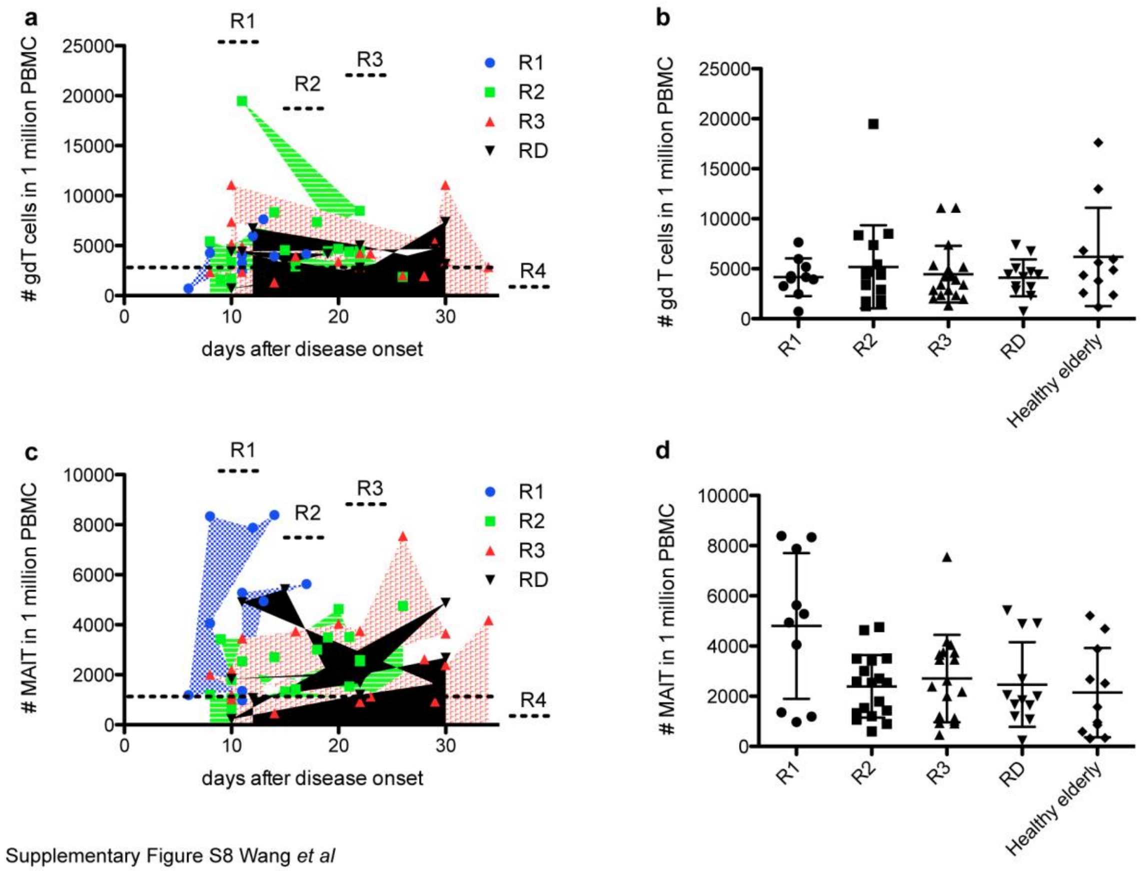


**Supplementary Fig. 8. Numbers of MAIT and γδ T cells in H7N9-infected patients.** The absolute numbers of (**ab**) γδ T cells and **(cd)** MAIT cells are shown across patients' groups during the disease course. (**bd**) The numbers of MAIT and γδ T cells in healthy elderly controls are shown.

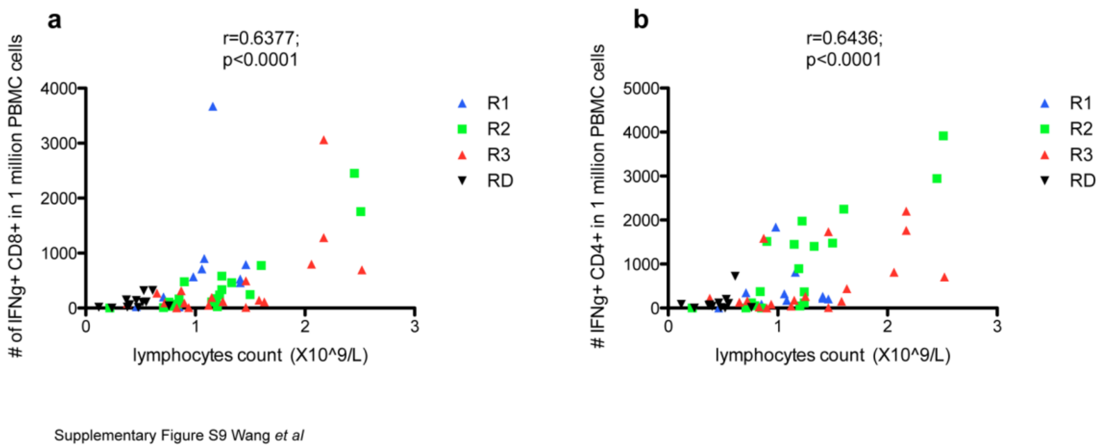


**Supplementary Fig. 9. Correlation between H7N9-specific (a) CD8$^{+}$ and (b) CD4$^{+}$ T cells and lymphocyte counts.** Analysis was performed by a non-parametric Spearman test.

Supplementary Table 1. Demographics of the A/H7N9 patient cohort.

| Group[a] | | Patient No. | Sex[b] | Age | Underlying medical disorders | Days from disease onset to: The initiation of oseltamivir | 1st treatment of Methylprednisolone | Admission | HLA subtype: HLA-A | HLA-A | HLA-B | HLA-B | Clinical outcome: Discharged after days of onset or death | hosptial stay length (days) |
|---|---|---|---|---|---|---|---|---|---|---|---|---|---|---|
| Group A (recovery outcome) | R1 | a73 | M | 53 | None | 5 | 5 | 6 | 2402 | 2402 | 1502 | 5401 | 14 | 8 |
| | | a107 | M | 47 | None | 5 | 5 | 7 | 0207 | 3303 | 1601 | 5409 | 17 | 10 |
| | | a49 | M | 68 | Hypertension II | 6 | No | 8 | 0203 | 1101 | 1301 | 4403 | 18 | 10 |
| | | a10 | M | 65 | Hypertension Urathritis | 4 | No | 8 | 0201 | 3201 | 0702 | 4001 | 18 | 10 |
| | R2 | a134 | F | 74 | Hypertension III, Coronary Heart Disease (NYHA IV), Diabetes II, Cholecystitis | 8 | No | 8 | 2601 | 3001 | 1302 | 3901 | 21 | 13 |
| | | a78 | M | 74 | None | 8 | 12 | 10 | 0207 | 2402 | 4601 | 4801 | 22 | 12 |
| | | a20 | F | 81 | Arhythmia | 5 | 6 | 6 | 1102 | 2601 | 1518 | 1518 | 23 | 17 |
| | | a9 | M | 67 | None | 5 | 4 | 9 | 0201 | 0207 | 1511 | 4002 | 23 | 14 |
| | | a130 | M | 67 | Diabetes II | 11 | 9 | 9 | 0201 | 2402 | 4501 | 4601 | 27 | 18 |
| | R3 | a79 | M | 78 | Hypertension II | 11 | 15 | 10 | 0201 | 1101 | 1501 | 5603 | 31 | 21 |
| | | a11 | F | 75 | Hypertension II, Coronary Heart Disease (NYHA IV), Diabetes | 8 | 9 | 9 | 3001 | 3101 | 1302 | 4006 | 33 | 24 |
| | | a12 | M | 62 | Hypertension | 9 | 5 | 8 | 0101 | 3001 | 1302 | 3701 | 35 | 27 |
| | | **Average** | | **67.7** | | **7** | **7.7** | **8** | | | | | | |
| Group B (fatal outcome) | RD | a131 | F | 79 | Arhythmia, Coronary Heart Disease (NYHA IV),Chronic Bronchitis , primary biliary cirrhosis | 6 | 6 | 8 | 301 | 1101 | 1302 | 3802 | | 56 |
| | | a22 | M | 58 | Hypertension | 9 | 8 | 12 | 2301 | 2601 | 3501 | 5512 | Death | 54 |
| | | a33 | M | 56 | None | 3 | 7 | 8 | 206 | 3101 | 4006 | 5601 | | 62 |
| | | a118 | M | 88 | Hypertension III, Coronary Heart Disease (NYHA IV), Diabetes II, Chronic bronchitis | 7 | 8 | 9 | 207 | 1101 | 3901 | 4601 | | 11 |
| | | **Average** | | **68.5** | | **6.3** | **7** | **10** | | | | | | |
